# Evolutionary conservation of the intrinsic disorder-based Radical-Induced Cell Death1 hub interactome

**DOI:** 10.1038/s41598-019-55385-3

**Published:** 2019-12-12

**Authors:** Lise Friis Christensen, Lasse Staby, Katrine Bugge, Charlotte O’Shea, Birthe B. Kragelund, Karen Skriver

**Affiliations:** REPIN and the Linderstrøm-Lang Centre for Protein Science, Department of Biology, University of Copenhagen, Copenhagen, DK-2200 Denmark

**Keywords:** Protein analysis, Proteins, Molecular biophysics

## Abstract

Radical-Induced Cell Death1 (RCD1) functions as a cellular hub interacting with intrinsically disordered transcription factor regions, which lack a well-defined three-dimensional structure, to regulate plant stress. Here, we address the molecular evolution of the RCD1-interactome. Using bioinformatics, its history was traced back more than 480 million years to the emergence of land plants with the RCD1-binding short linear motif (SLiM) identified from mosses to flowering plants. SLiM variants were biophysically verified to be functional and to depend on the same RCD1 residues as the DREB2A transcription factor. Based on this, numerous additional members may be assigned to the RCD1-interactome. Conservation was further strengthened by similar intrinsic disorder profiles of the transcription factor homologs. The unique structural plasticity of the RCD1-interactome, with RCD1-binding induced α-helix formation in DREB2A, but not detectable in ANAC046 or ANAC013, is apparently conserved. Thermodynamic analysis also indicated conservation with interchangeability between Arabidopsis and soybean RCD1 and DREB2A, although with fine-tuned co-evolved binding interfaces. Interruption of conservation was observed, as moss DREB2 lacked the SLiM, likely reflecting differences in plant stress responses. This whole-interactome study uncovers principles of the evolution of SLiM:hub-interactions, such as conservation of α-helix propensities, which may be paradigmatic for disorder-based interactomes in eukaryotes.

## Introduction

Most functional proteins fold into well-defined structures. However, 30–50% of eukaryotic proteins contain large regions of intrinsic disorder (ID)^1,2^ allowing function without well-defined folds^3–8^. Intrinsically disordered proteins (IDPs) and -regions (IDRs) often participate in protein-protein interaction networks (interactomes), which govern key functions such as transcription and cell-cycle regulation^9–12^. Accordingly, transcription factors (TFs) contain high fractions of functionally essential IDRs^13,14^. IDPs evolve faster and are more permissive to substitutions than folded proteins^15,16^. However, sites within IDRs with secondary structure propensities are evolutionary more constrained than sites within secondary structures^17^. Such regions may coincide with short linear motifs (SLiMs), originally discovered as islands of taxonomic conservation within rapidly evolving regions^18,19^. SLiMs are regularly gained and lost during evolution making them useful tools for determining clade-specificity^19,20^. While SLiMs are not limited to IDRs, molecular recognition features (MoRFs) represent a concept developed for disorder-based interactions. MoRFs are structure-prone regions located within long IDRs, which may undergo disorder-to-order transitions upon binding^21^. Evolutionary studies of the p53 TF family have shown that MoRFs are more conserved than their surrounding regions^22^. Although several bioinformatics studies have addressed the evolution of IDPs, SLiMs, and MoRFs, most of these studies lack biophysical support and perspectives on the whole interactome.

**Figure 1:**
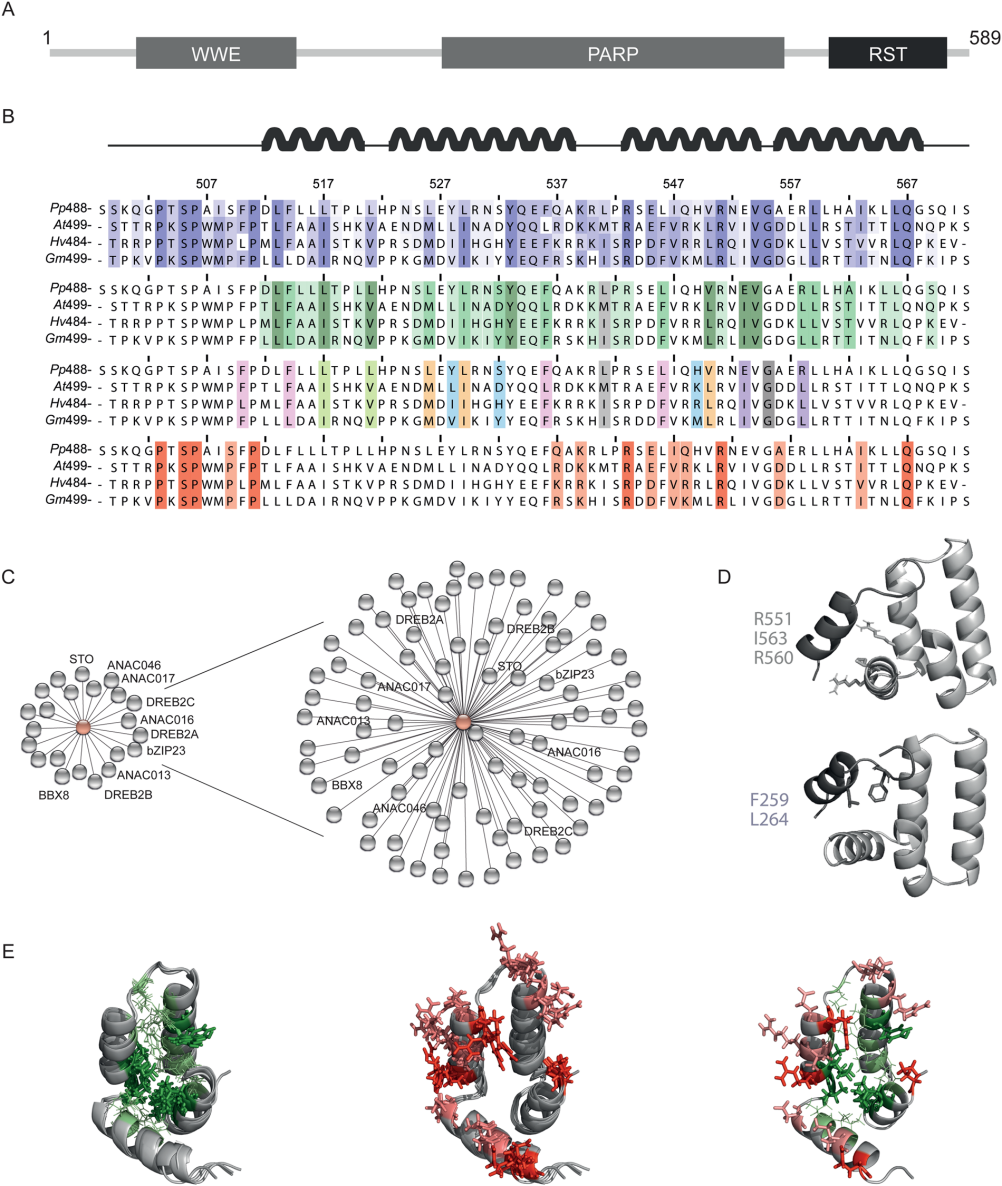
RCD1 structure and interactions. (**A**) Domain structure (WWE, PARP, and RST domains) of *At*RCD1. (**B**) Sequence alignment of *At*RCD1-RST with the most related RST domain from barley, soybean, and moss. α-helix positions in *At*RCD1-RST are shown at the top. Top alignment: Conservation of residues with colouring in accordance with percentage identity (Jalview; darker blue is more conserved). Middle top alignment: Colouring in accordance with number of contacts (6 Å cut-off) to other structural elements (helix 1–4 or loop 1–2) in the *At*RCD1-RST structure (PDB 5OAO). Dark green is three, green two, and light green one contact. The β_3_-position of the α_L_-β_4_ loop motif is grey. Middle bottom alignment: Residue groups that are in contact in the *At*RCD1-RST structure (PDB 5OAO;5N9Q) and appear to co-evolve in same colour. The β_3_-position of the α_L_-β_4_ loop motif is grey, Gly555 in dark grey. Bottom alignment: Conserved residues that cannot be explained by fold conservation are highlighted. Conserved residues are red, while conservative substitutions are light red. **(C)** Left: TF-interactome of *At*RCD1 from the STRING database. Names of TFs, which have been shown to bind the RST domain^30^, are shown. Right: Expanded *At*RCD1:TF interactome predicted based on the expanded SLiM [ED].{1,2}[^RK][YF].{1,4}[^RK][DE]([LIVMF]|.L). **(D)** Structure of the *At*RCD1 RST domain (light grey; PDB 5OAO) in modelled complex with *At*DREB2A(255–272) (dark grey; PDB 5OAP)^25^. *At*RCD1-RST key residues (R551, R560, I563) for *At*DREB2A(255–272) binding and the α-helix stabilizing hydrophobic staple motif residues, F259 and L264, of *At*DREB2A(255–272), are highlighted. **(E)** NMR solution structure of *At*RCD1-RST (light grey; PDB 5N9Q) and models (light grey) of the barley, soybean, and moss RST domains generated using I-TASSER^70^. Left: Superposition of structure and models with residues having three or two contacts to other structural elements in dark green sticks and green lines, respectively. Middle: Superposition of structure and models with conserved residues not explained by fold conservation shown as sticks. Fully conserved residues are red while positions with conservative substitutions are light red. Right: Structure of *At*RCD1-RST (light grey; PDB 5N9Q) with same residue highlights as the superpositions (left and middle).

The plant hub-protein Radical-Induced Cell Death 1 (RCD1) represents a suitable model for studies of interactions between folded hub domains and SLiMs in disordered targets, and allows translation from *in vitro* to the organismal level^23^. RCD1 is a member of the plant-specific Similar to Rcd One (SRO) family. SRO proteins contain a Poly (ADP-Ribose) Polymerase (PARP) domain and an RCD1-SRO-TAF4 (RST) domain^24^, and some also have a WWE domain (Fig. 1A). *Arabidopsis thaliana* RCD1 (*At*RCD1, with *At* referring to the species-specific origin), uses its helical RST domain (Fig. 1B), a member of the αα-hub family^25^, for interactions with many TFs^26^. *At*RCD1 plays roles in hormone signaling, responses to reactive oxygen species and other abiotic stress factors, immunity and development^26–29^. It interacts with numerous TFs (Fig. 1C)^26,30^, including Dehydration-Responsive Element-Binding protein (DREB) 2 A, implicated in abiotic stress responses^31^. Recent studies suggest that *At*RCD1 negatively regulates its interaction partners. Thus, downregulation of *At*RCD1 or loss of the RCD1-interacting site of DREB2A is required for proper DREB2A function under stress conditions^32^, and inactivation of RCD1 resulted in increased expression of Arabidopsis No-apical-meristem, Arabidopsis transcription activation factor, Cup-shaped cotyledon (ANAC)013 and ANAC017-regulated genes from the mitochondrial dysfunction stimulon^33^.

Recently, the consensus motif [ED].{1,2}[^RK][YF].{1,4}[^RK][DE]L was identified as the RCD1-binding SLiM (hereafter referred to as RBS) positioned in IDRs of RCD1 target TFs^30^. Interestingly, different structures, containing this motif, may be formed in the TFs upon association with the RST domain of RCD1. While α-helix is induced in DREB2A (Fig. 1D), no helical structure has been observed in ANAC046 and ANAC013 upon complex formation^30^ suggestive of structural plasticity in the RCD1-interactome.

In this study, the evolution of the RCD1:TF interactome was analyzed. This revealed evolutionary conservation of RCD1, the RBS and the TF order-disorder patterns in land plants over a period of 480 million years. Both cross-species interactions and molecular co-adaptation in high-affinity RCD1:TF interactions were observed. Interruption of conservation in moss DREB2, which does not contain the SLiM, may reflect differences in plant stress responses. Our whole-interactome approach, spurred by ID profiles and motif based interactions in a molecularly intriguing interactome, is readily transferrable to other ID-based interactomes.

## Results

### Taxonomic conservation of the SRO family

**Figure 2:**
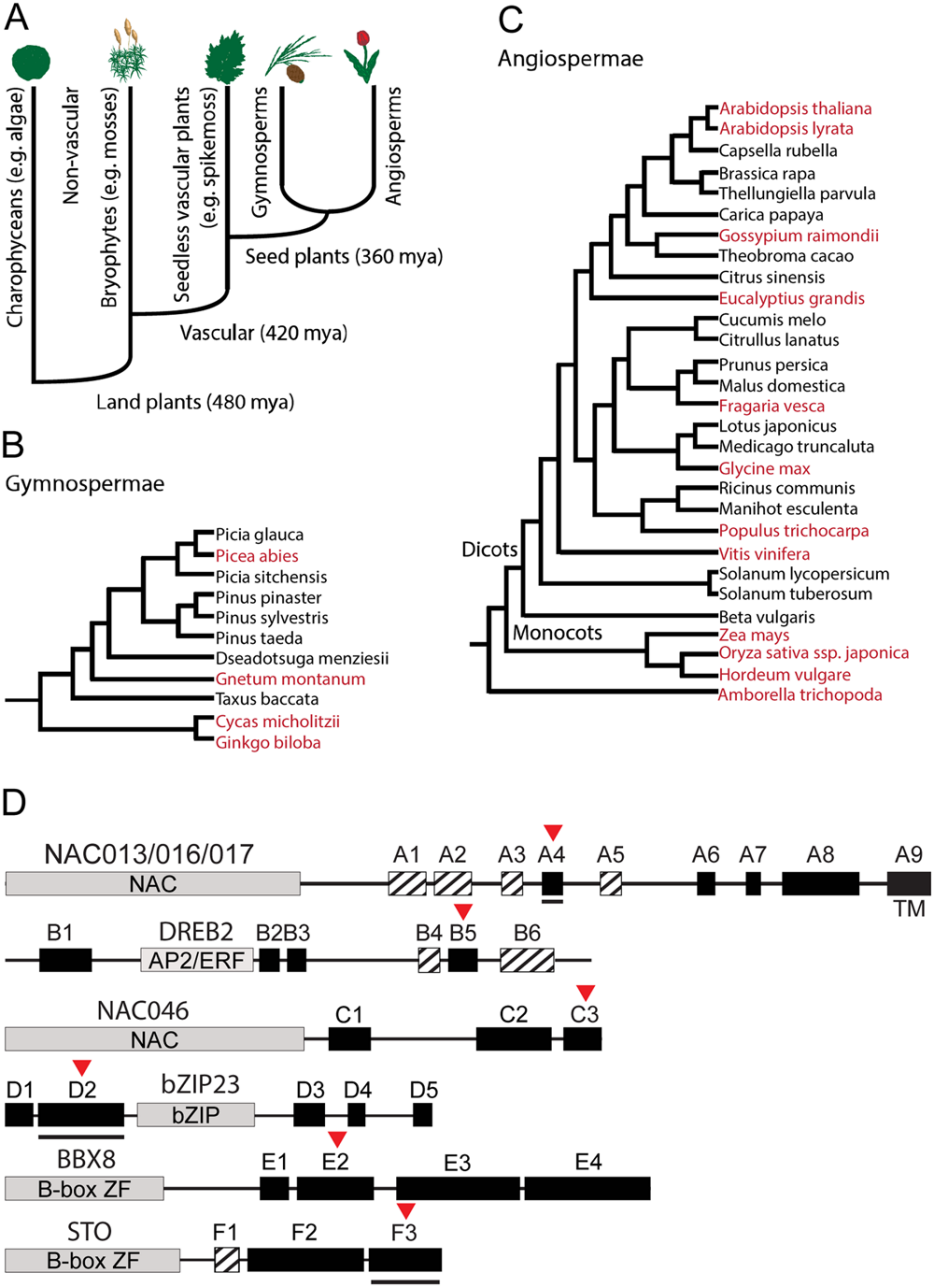
Phylogenetic relationships of plants analysed for RCD1-interacting motif in transcription factors with the domain structure shown. **(A)** Evolution of the plant kingdom showing splitting of land plants into nonvascular and vascular plants, and further into seedless and seed plants. **(B)** Phylogenetic relationship of gymnospermea (non-flowering) species (modified from PLAZA^34,35,72^). **(C)** Phylogenetic relationship of angiospermea (flowering) species (modified from PLAZA^34,35,72^). Species included in this study are highlighted in red. **(D)** Domains and conserved sequence patterns for NAC013/016/017, DREB2, NAC046, bZIP23, BBX8 and STO TFs. The DBDs are grey, sequence patterns conserved in land plants are black, while sequence patterns conserved specifically in angiosperms are hatched. The positions of the RBS are marked by a red arrow. Black lines below boxes indicate regions where ELMs overlap with the SLiM. TM: transmembrane region. The figures are not drawn to scale.

Ortholog identification, which is generally accepted as a proxy for identification of genes with similar functions in different species^34^, was used for analysing the evolution of the SRO:TF interactome. Sequences were obtained from PLAZA, developed specifically for comparative genomics in plants with many paralogs^34,35^. Since SRO proteins have been identified only in land plants, these were the focus of this study (Fig. 2A; Supplementary Table S1)^36,37^. The moss *Physcomitrella patens* was the only non-vascular plant included. *Selaginella moellendorffii* (Fig. 2A; Supplementary Table S1) represents lycophytes, whereas *Cycas micholitzii*, *Ginkgo biloba*, *Gnetum montanum* and *Picea abies* represent gymnosperms (Fig. 2B). In addition, *Amborella trichopoda*, a living ancestor of the sister lineage to all other living angiosperms, *Oryza sativa ssp. japonica*, *Zea mays*, and *Hordeum vulgare* representing different monocot clades (Fig. 2C), and eight dicots were included. Comparison of *Arabidopsis thaliana* and *Arabidopsis lyrata* reveals recent evolutionary changes, whereas the other dicots *Eucalyptus grandis, Populus trichocarpa, Fragaria vesca, Vitis vinifera, Gossypium Raimondii*, and *Glycine max* were selected to represent diversity. SRO proteins have been identified in all of these species, except six, which were analysed here (Supplementary Table S2). Several sequences obtained using Integrative Orthology Viewer were incomplete, and only sequences with PARP and RST domains were used for further analysis. The number of identified SRO proteins varied from none for *Gnetum montanum* to four for *Amborella trichopoda, Zea mays*, and *Ginkgo biloba*, and *Gnetum montanum* was, therefore, not included in this study (Fig. 2B; Supplementary Table S2). Searching pico-PLAZA 2.0, which represents eukaryotic microorganisms, confirmed that SRO proteins are specific to land plants.

The structure of *At*RCD1-RST consists of four α-helices organized in an L-glove and with a super-secondary αα-hairpin structure as its base formed by helices 2 and 3^25^. Molecular modelling using the *At*RCD1-RST solution structure as template indicated that the RST domain structure is conserved from moss to different flowering plants (barley and soybean). From sequence alignments of Arabidopsis, moss, barley and soybean RST domains in combination with analysis of the tertiary structure of the *At*RST domain^25,33,38^ it is clear that the residues that make up the central hydrophobic core responsible for the RST-fold are highly conserved (Fig. 1B, middle top). Here, a LIVYLIV motif (*At*: L-x_3_-I-x_3_-V-x_11_-Y-x_16_-L-x_2_-I-V) is responsible for the central core at the closed end of the helix 1–3 assembly (Fig. 1B, middle top;1E, left), and the fold is further supported by a 3xPhe-1xLeu motif at the open end and conserved hydrophobic residues of helix 4 contacting the LIVYLIV network (Fig. 1B, middle top; 1E, left). The sequences align without any insertions or deletions and several residue positions that are in contact in the *At*RST structure have co-evolved across the species (Fig. 1B, middle bottom). The stabilizing β_3_-position of the α_L_-β_4_ loop motif of the fundamental αα-hairpin super-secondary structure^39^ is maintained across the species but alternates between different, favoured residues for this motif position (Met541 in Arabidopsis, Leu in moss, Ile in soybean and barley). Gly555 (residue numbering relative to *At*RCD1), the one-residue loop responsible for the angle between helix 3 and 4, is likewise conserved across the species. Taken together, these findings indicate that the RST fold is highly conserved across the species.

It is also clear that there are conserved residues that are not immediately explained by structural conservation (Fig. 1B, bottom). Besides a Pro-motif in the disordered N-terminus, these mainly localize to helix 3 and 4 and are mostly Arg (543, 551) or Arg/Lys (537, 539, 548), but also Glu/Asp (545), Asp (556), Ile/Val (547, 563) and Gln (567). Nonetheless, Lys/Arg_537_, Lys/Arg_548_ and Asp_556_ are charge neutralized by substitutions with Gln, Gln and Ala, respectively, in moss. When plotted onto the structure of *At*RST and the models, these residues localize to the rim of the hydrophobic L-glove pocket of the structure where DREB2A binds to the hub (Fig. 1E, middle), which could indicate that they influence binding affinity and specificity.

### Identification of TF orthologs and inparalogs

For analysis of evolutionary conservation of the RCD1-interactome, orthologs and inparalogs of the ten Arabidopsis TFs with a functional SLiM^30^ were identified and aligned. Since the Arabidopsis NAC proteins ANAC013, ANAC016 and ANAC017 (with A in ANAC referring to the Arabidopsis origin) and the Arabidopsis DREB2 proteins *At*DREB2A, *At*DREB2B and *At*DREB2C, respectively, are closely related only one multiple protein sequence alignment was created for each homology group. The sequences generally align well in the DNA-binding domains (DBDs), whereas sequence variation outside these is consistent with ID^30,40^. However, conserved sequence patterns, which can be regarded as homolog signatures, were found throughout the IDRs (Fig. 2D).

**Figure 3:**
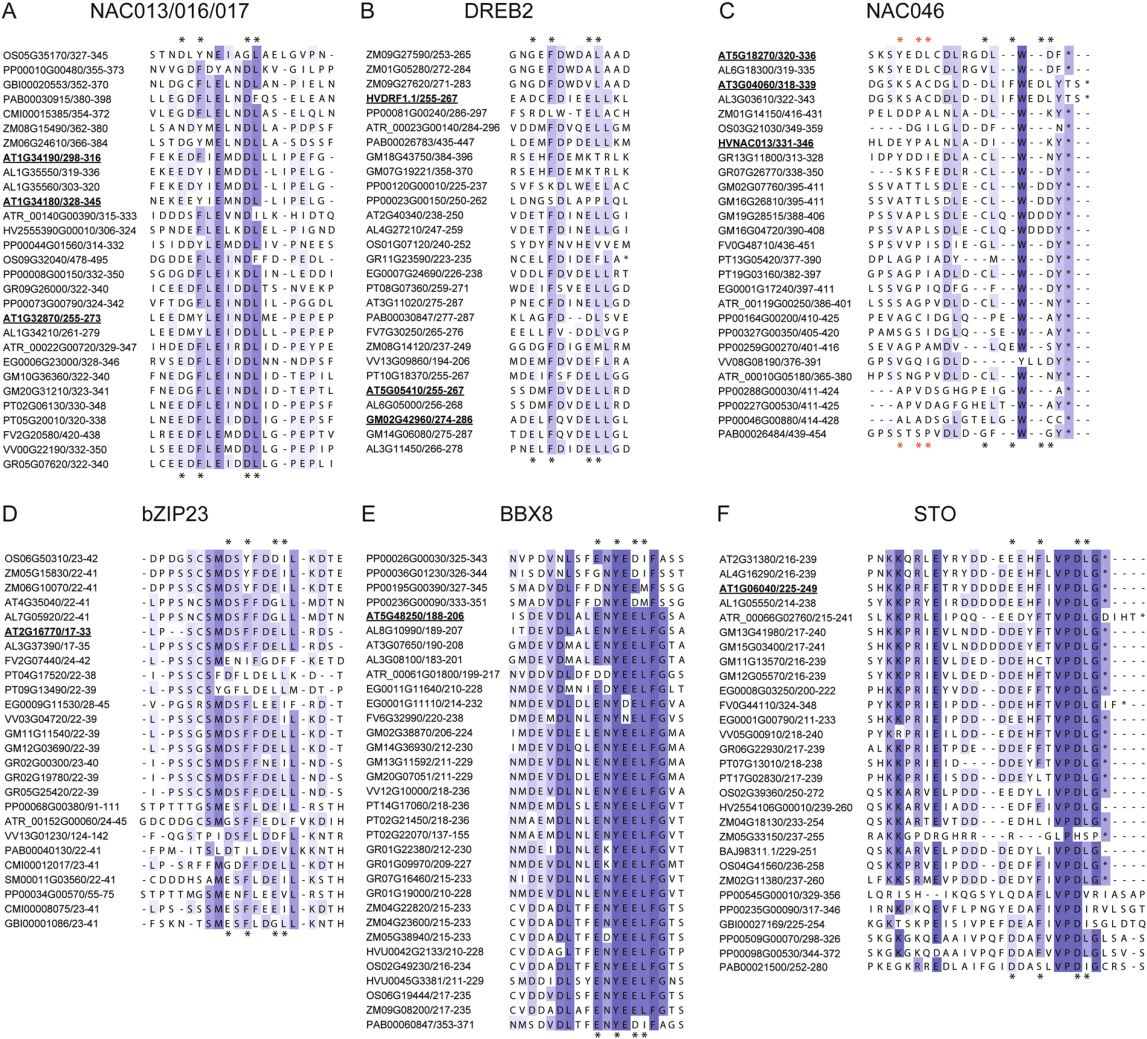
Conservation of the RBS. **(A**–**F)** Sequence alignments based on the experimentally verified RBS from the Arabidopsis TFs ANAC013 (AT1G32870), ANAC016 (AT1G34180), ANAC017 (AT1G34190), *At*DREB2A (AT5G05410), *At*DREB2B (AT3G11020), *At*DREB2C (AT2G40340), ANAC046 (AT3G04060), *At*bZIP23 (AT2G16770), *At*BBX8 (AT5G48250), and *At*STO (AT1G06040)^30^ with homologs from different species. The gene names of experimentally verified binders of *At*RCD1-RST are underlined. The aligned regions do not represent the exact peptides used in the experimental studies (Table 2). The figure shows the names without the prefix A or *At*, specifically referring to NAC TFs of Arabidopsis origin, since sequences from many different plant species are shown. The amino acid residues are coloured according to the degree of conservation (Jalview; darker blue is more conserved). For all alignments, SLiM residues are marked with black asterisks in the top and bottom of the alignment based on the experimentally verified binders from each family^30^. For ANAC087 (At5g18270), the alternative putative SLiM variant is marked with red asterisks. The sequences were obtained from the PLAZA platform^34,35,72^ for the following species: *Physcomitrella patens* (PP), *Selaginella moellendorffii* (SM), *Amborella trichopoda* (ATR), *Oryza sativa ssp. Japonica* (OS), *Zea mays* (ZM), *Arabidopsis lyrata* (AL), *Arabidopsis thaliana* (AT), *Eucalyptus grandis* (EG), *Fragaria vesca* (FV), *Glycine max* (GM), *Gossysium raimondii* (GR), *Populus trichocarpa* (PT), *Vitis vinifera* (VV), *Ginkgo biloba* (GBI)*, Cycas micholitzii* (CMI)*, Gnetum montanum* (GMO) and *Picea abies* (PAB). The sequences of the plant species *Hordeum vugare* (HV, BAJ or AAO) were obtained by BLAST homology searches. Extended alignments including non-binders can be found in Supplementary Fig. S1.

The alignment of the NAC013/016/017 TFs revealed a conserved DBD and various C-terminal regions corresponding to disordered transcriptional activation domains^40^. Nine short conserved regions (A1–9) were apparent with the RBS mapping to A4 (Fig. 2D). The SLiM was identified in three of the four moss, the three gymnosperm and all of the dicot homologs, and for monocots in one maize, rice (reverse orientation) and barley homolog (Fig. 3A). Although it was lacking from one of the rice and Amborella sequences, these contained a motif with isoleucine substituting leucine conferring to the motif [ED].{1,2}[^RK][YF].{1,4}[^RK][DE][LI]. A search in the eukaryotic linear motif (ELM) database revealed a docking motif for PIKK kinases in 15 dicot homologs, which overlaps with the SLiM.

In the DREB2 TFs, the DBD is flanked by IDRs with six conserved regions (B1–6; Fig. 2D). In addition to being present in the *At*DREB2s^30^, the SLiM was found in the Amborella ortholog, one of the two Norway spruce orthologs as well as in most of the dicot orthologs (Fig. 3B). It was not identified in the DREB2 TFs from moss, and for monocots, it was only found in barley, explaining the inability of rice *Os*DREB2A to interact with *Os*SRO1c^41^. For several homologs, most of the core residues are present, and in three cases [DE].L terminates the motif suggestive of motif expansion to [ED].{1,2}[^RK][YF].{1,4}[^RK][DE](L|.L). Thus, although the existence of the SLiM is unlikely for non-vascular DREB2s, it is conserved in seed plants.

In the NAC046 TFs, the SLiM is located in the most C-terminal conserved region, C3 (Fig. 2D). It is conserved in the ortholog most closely related to ANAC046, AL3G03610, but it is not generally conserved (Fig. 3C). A tryptophan residue appears to be conserved among the NAC homologs, but Trp332 in ANAC046 was shown to have a less significant effect on RCD1-binding than the RBS-defining residues. Therefore, the conserved tryptophan pattern may reflect the presence of an overlapping activation motif of transcription as previously suggested^42^. The RBS is present in one of the moss homologs and in homologs from cotton and eucalyptus, but located in the region between C2 and C3 (Supplementary Fig. S1). In this region, ten sequences differ from the SLiM only by the absence of the acidic residue in the first SLiM position, thus conferring to. {1,2}[^RK][YF][ED]L. In total, sequence patterns identical or similar to the SLiM are observed in 13 of 27 NAC046 homologs within or close to C3.

For the bZIP23 TFs with five conserved regions, the SLiM maps to D2 (Fig. 2D) in six of the plant species, and [ED].{1,2}[^RK][YF].{1,4}[^RK][DE]([LI]|.L) is present in bZIP23 TFs from several species including moss and spikemoss, whereas other homologs have motifs described by.{1,2}[^RK][YF].{1,4}[^RK][DE]L or [ED].{1,2}[^RK][YF].{1,4}[^RK][DE][FIMV] (Fig. 3D). Thus, the SLiM and single residue variants were identified in all species except Norway spruce. The phosphorylation site ELM MOD_CK1_1 maps to D2 of the bZIP23 TFs.

Four conserved regions were identified for the BBX8 TFs with the SLiM mapping to E2 (Fig. 2D). In all dicot BBX8 TFs, the SLiM is bi-directional because of conservation of an additional leucine (Fig. 3E). It is lacking from three moss homologs and the Norway spruce homolog which nonetheless have the expanded SLiM [ED].{1,2}[^RK][YF].{1,4}[^RK][DE][LIM], suggestive of conservation across land plants.

The SLiM maps to F3 in the STO TFs (Fig. 2D). It is present in half of the moss STOs, but missing from the gymnosperm STOs (Fig. 3F). However, several of these contain the expanded SLiM [ED].{1,2}[^RK][YF].{1,4}[^RK][DE][LIV]. The N-terminal conserved region of the bipartite F3 region is specific for angiosperm STO TFs, whereas a second conserved region is located at the C-termini in mosses and gymnosperms (Supplementary Fig. S1). The degradation ELM DEG_COP1_1 is present in most of the sequences and overlaps with the SLiM.

### Non-binding motifs

*At*MYB91 and *At*DREB2A contain a motif that meets the criteria of the RBS, but is not able to bind RCD1-RST^30^. In *At*MYB91, the non-binding motif is present at the N-terminal border of a conserved region in five additional MYB91 homologs, but for the rest of the homologs, the SLiM is absent. Similarly, the non-binding motif of *At*DREB2A is located in a non-conserved region. Therefore, and importantly, non-binding SLiMs differ from binding-competent SLiMs by being non-conserved (Supplementary Fig. S1). Thus, conservation can be used as an additional filter for the identification of functional RBS regions.

### Conservation of ID patterns and MoRFs

**Figure 4:**
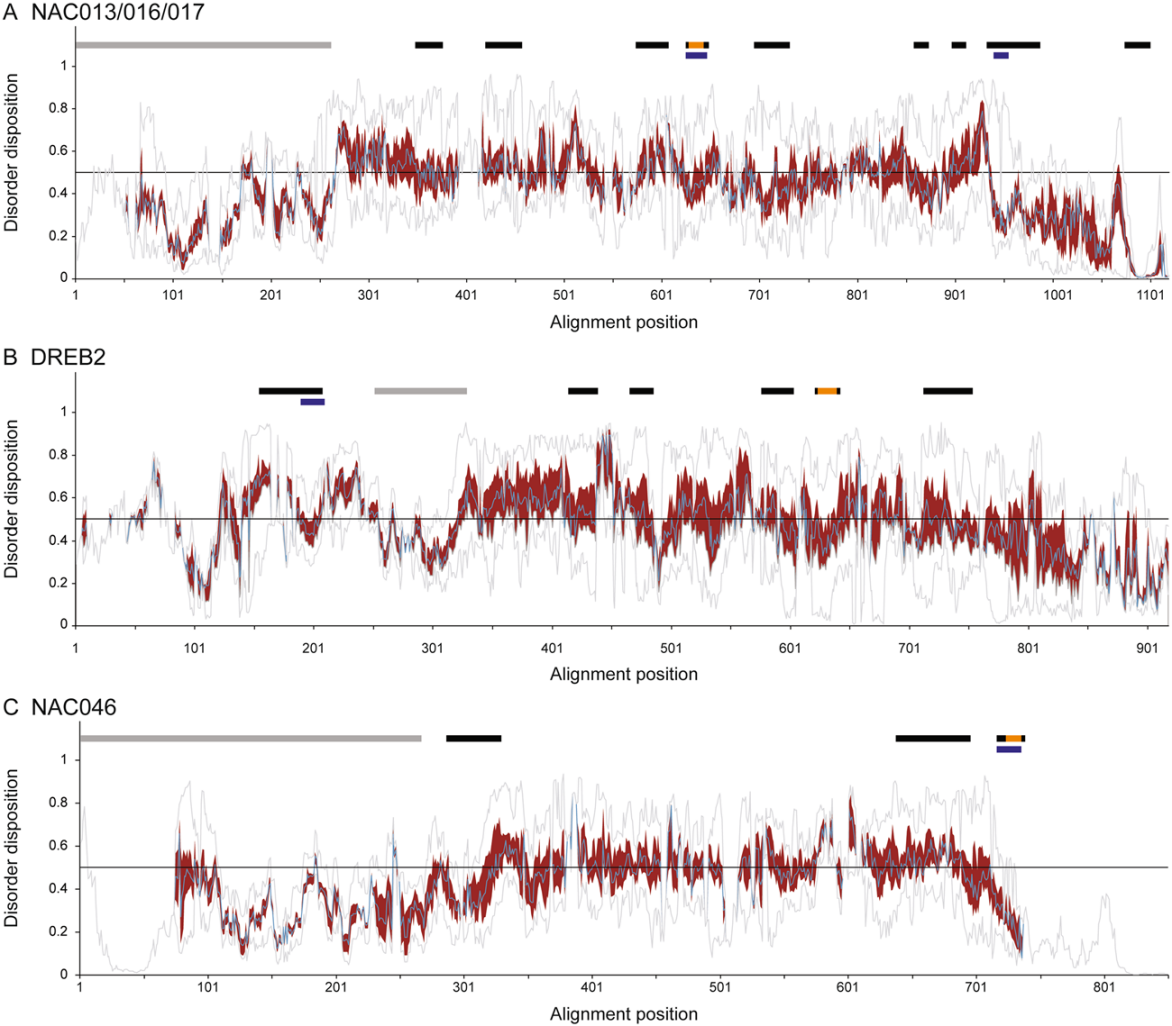
ID and MoRF predictions for RCD1-interacting TFs**. (A**–**C)** IUPRED ID predictions for the NAC013/016/017, DREB2 and NAC046 homologs made from Jalview alignments of the sequences included in Fig. 3. The IUPRED scores of the homologues protein sequences are plotted for each alignment position by the minimum and maximum (grey curves), the median (blue curve) and the interquartile range as the (red) area between the 1. and 3. quartiles. The threshold of 0.5 for ID is marked by a line. Blue bars represent positions for which MoRFs were predicted for more than 50% of the aligned homologs using MoRFpred. Black bars show the positions of conserved sequence motifs. Orange and grey bars show the positions of the RBS and DBD, respectively.

Disorder-order patterns have been suggested to be more constrained than sequences^43^, and for different NAC sub-groups the ID profiles are well conserved^44^. The ID profiles of the NAC013/016/017, DREB2 and NAC046 TFs were analyzed using IUPRED, which allows disorder prediction for multiple sequences^45^. In the TFs, the DBDs have similar ID-profiles reflecting structured domains (Fig. 4A–C). In contrast, the profiles vary for the IDRs, although similarities were also apparent. The RBS maps to a dip in the NAC013/016/017, DREB2 and NAC046 disorder profiles suggestive of local structure propensities. The long disordered NAC046 C-termini with sparse sequence motifs (Fig. 2D) and secondary structures^42^ provide a conserved flexible platform for interactions mediated by the RBS at the very C-termini. About half of the conserved regions of the three TF groups also map to dips in the disorder profiles. The TFs were also analyzed for MoRF conservation. For the NAC013/016/017 and the NAC046 TFs, a MoRF was predicted for the RBS region in more than 50% of the TFs. However, MoRFs were not generally conserved for the motif regions. To conclude, similarities were observed in the ID-profiles of the long IDRs with the RCD1-binding SLiM mapping to regions with local structure propensity.

### Expanding the RBS

The results indicate that the RBS, [ED].{1,2}[^RK][YF].{1,4}[^RK][DE]L, is conserved among the NAC013/016/017, DREB2, STO and BBX8 homologs with some exceptions. It is missing from the moss DREB2 TFs, whereas the lack of spikemoss homologs and SLiMs may be explained by the quality of the sequence data for this organism. For the NAC046 and bZIP23 TFs, the SLiM is only present in a few species, and it is not completely conserved for any of the TF groups. However, most of its core residues are conserved, and three alternative SLiM versions (variant 1–3, referred to as RBS-V1 - V3) (Table 1) are apparent. These differ from the original RBS only at one position and are in accordance with the SLiM-discriminatory features of a central aromatic residue, pI value below 4.5^30^ and conserved location in the parent protein. RBS-V1 lacks the acidic residue in the first SLiM position, in RBS-V2 the C-terminal leucine is substituted with phenylalanine, isoleucine, methionine or valine, and in RBS-V3 an additional residue is inserted before the C-terminal leucine. The original SLiM was identified in 99 homologs and is more frequent than the alternative versions. However, variant 1 is most frequent among the NAC046 TFs, while variant 2 and 3 are most frequent among the bZIP23 TFs. The frequent alternative motifs are all described by the expanded motif [ED].{1,2}[^RK][YF].{1,4}[^RK][DE]([LIVMF]|.L). This SLiM, which would increase the number of TFs in the RCD1-interactome from 21 to 166 (Fig. 1C; Supplementary Table S3), is found across all lineages of land plants indicating conservation of the pattern. Although DREB2 and STO TFs were identified in microorganisms, these TFs lack the SLiM in accordance with a main role of the SLiM being RCD1-binding.

**Table 1:** The original RBS and alternative SLiM versions identified in this study and named RBS-V1 – RBS-V3.

TF group		NAC013/016/017	DREB2A DREB2B DREB2C	STO	NAC046	BBX8	bZIP23
RBS	[ED].{1,2}[^RK][YF].{1,4}[^RK][DE]L	23/29	15/28	22/30	5/27	26/33	8/27
RBS-V1	.{1,2}[^RK][YF].{1,4}[^RK][DE]L	1	1	0	12	0	3
RBS-V2	[ED].{1,2}[^RK][YF].{1,4}[^RK][DE][FIMV]	5	6	3	1	4	12
RBS-V3	[ED].{1,2}[^RK][YF].{1,4}[^RK][DE].L	0	3	0	1	0	13

The six plant TF groups containing members, which have been experimental verified to bind to RCD1-RST domains^30,42^, are shown at the top. To the left of the stroke: Number of homologs containing the RCD1-binding SLiM. To the right of the stroke: Number of identified homologs. The number of occurrences of the three alternative SLiM versions in TFs without the consensus RBS are also shown.

### Experimental verification of the expanded RBS

**Figure 5:**
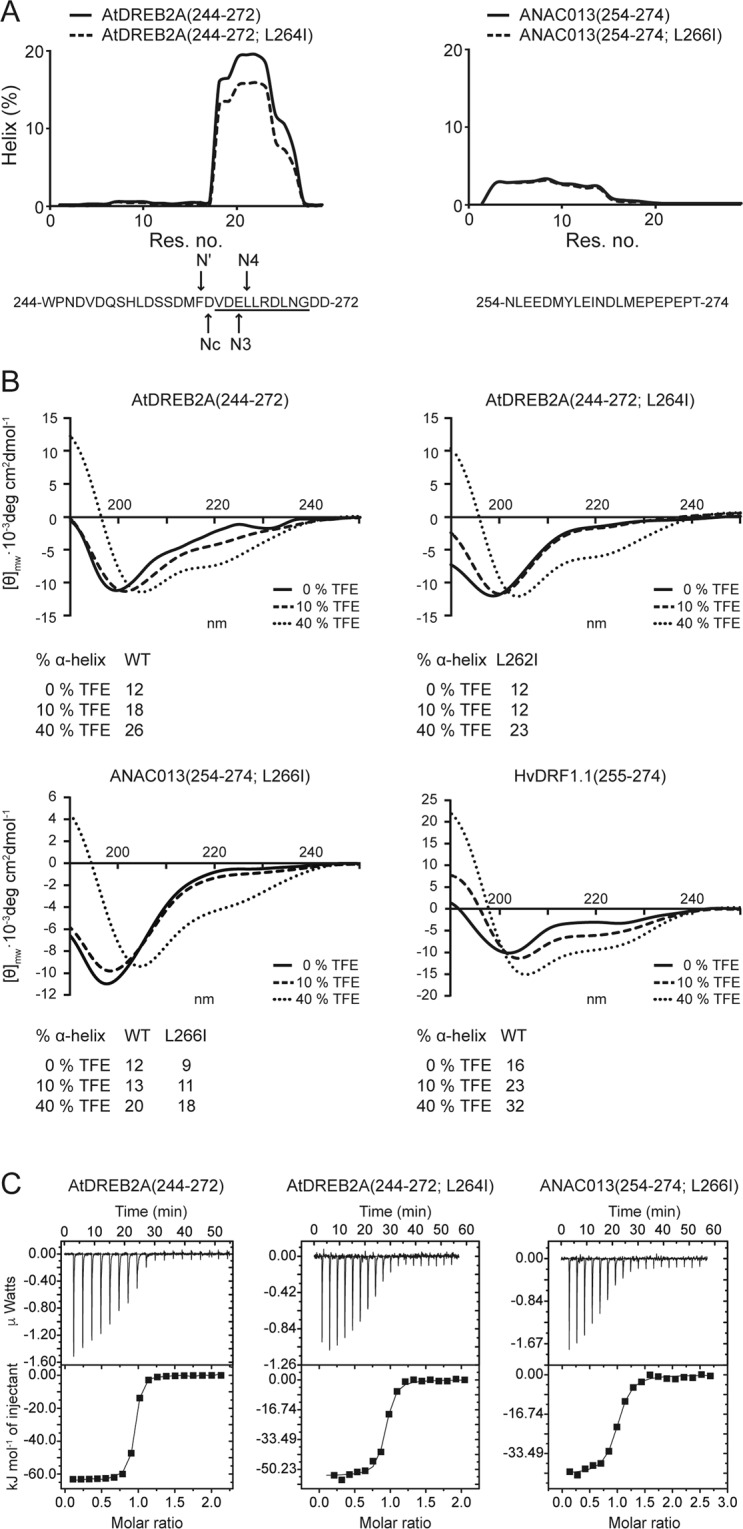
Experimental verification of SLiM variants**. (A)** Left: Helicity per residue predicted by Agadir for *At*DREB2A(244–272) and *At*DREB2A(244–272; L264I) (top) and sequence of *At*DREB2A(244–272) (bottom) with the N’ and N4 residues of the hydrophobic staple motif marked and the α-helix formed in complex with RCD1 underlined. Right: Helicity per residue predicted by Agadir for ANAC013(254–274; L266I). **(B)** Far-UV CD spectra of 15‒20 µM TF peptide as indicated above the spectra in 10 mM Na_2_HPO_4_/NaH_2_PO_4_, pH 7.0, and 0–40% (v/v) TFE. Data for ANAC013(254–274) has been reported previously^30^. (**C**) Representative ITC measurements, here shown for the RCD1-RST(499–572) interactions with *At*DREB2A(244–272), *At*DREB2A(244–272;L264I), and *At*ANAC013(254–274;L266I). The *At*ANAC013(254–274):*At*RCD1-RST(499–572) interaction was published previously^30^. *At*RCD1-RST(499–572) was titrated into the TF fragments. In each panel, the upper portion shows baseline-corrected raw data from the titration, and the lower portion shows the normalized integrated binding isotherms together with the fitted binding curves. The data were fitted to a “one set of sites” binding model. Parameters obtained from the non-linear fits are presented in Table 2.

RBS-V2 (Table 1) with isoleucine instead of leucine in the last SLiM position is found frequently among the TF homologs (Fig. 3). Since isoleucine destabilizes α-helices, this substitution could affect RCD1-binding of the NAC and DREB2 TFs differently, as they likely differ in their bound structure^30^. α-helical conformation was predicted for residues 261 to 270 of *At*DREB2A(244–272), and the Leu264 to Ile substitution resulted in only a slight overall decrease in helical propensity (Fig. 5A). A similar observation was made by circular dicroism (CD) spectroscopy in studies using trifluoroethanol (TFE) to probe for helical propensity (Fig. 5B). In buffer, a low population of α-helix was measured for both peptides, but addition of TFE to 10% had a slightly larger α-helix inducing effect on *At*DREB2A(244–272) than on *At*DREB2A(244–272; L264I). At 40% TFE, both spectra displayed the characteristics of α-helix, moving the minima towards 222 and 208 nm (Fig. 5B) indicating that the isoleucine variant is able to form α-helix to a similar extent as the wildtype peptide. For ANAC013(254–274)^30^ and ANAC013(254–274;L266I) the predicted level of α-helical conformation was low (Fig. 5A), and CD spectra suggested that the two peptides contain similar levels of α-helical structure (Fig. 5B).

Isothermal titration calorimetry (ITC) was used to analyse how Leu→Ile substitution affects binding to RCD1. *At*DREB2A(244–272) has high affinity (*K*_*d*_ 16 nM) for *At*RCD1-RST(499–572) and was not significantly affected by the change of Leu264 to Ile (Table 2; Fig. 5C). This is in accordance with structure analysis indicating that Asp260 forms the N-cap (first α-helix position) of the *At*DREB2A α-helix, which contains a hydrophobic staple motif^46^ with N’ and N4 interacting and stabilizing the α-helix by strengthening capping (Figs. 1D; 5A). In addition to Leu and Ile, Phe and Val, which are frequent in position N4 (Figs. 3B; 5A), also mediate α-helix stabilization by staple motif formation^46^ suggestive of evolutionary conservation of coupled folding and binding for DREB2 association with RCD1. Leu266 to Ile substitution in ANAC013(254–274) resulted in an approximately 20-fold decrease in affinity (*K*_*d*_ 223 nM) (Fig. 5C; Table 2), suggesting that β-branching may result in steric hindrance in the RCD1-ANAC013 complex. However, both the *At*DREB2A and the ANAC013 variants bound RCD1-RST(499–572) strongly, confirming that isoleucine, and other hydrophobic residues, are allowed at this last SLiM position, as suggested by the SLiM variants (Table 1).

**Table 2:** Thermodynamic analysis by ITC of interactions between RCD1-RST domains and different TFs.

TF	RCD1	*K*_*d*_ (nM)	*N*	Δ*H* (kJ/mol)	−TΔ*S* (kJ/mol)	Δ*G* (kJ/mol)	Syringe
*At*DREB2A(244–272)	*At*RST(499–572)	16 ± 1.4	0.90 ± 0.00	−63.3 ± 0.2	18.7	−44.6	RST
*At*DREB2A(244–272;L264I)	*At*RST(499–572)	39 ± 7.6	0.89 ± 0.01	−53.8 ± 0.7	11.5	−42.5	RST
ANAC013(254–274)^a^	*At*RST(499–572)	9 ± 4	0.80 ± 0.01	−45.0 ± 0.8	−0.6	−45.6	RST
ANAC013(254–274;L266I)	*At*RST(499–572)	223 ± 29	0.95 ± 0.01	−42.3 ± 0.5	4.3	−38.0	RST
ANAC087(315–335)	*At*RST(499–572)	1751 ± 625	0.93 ± 0.01	−15.9 ± 1.6	−16.9	−32.9	ANAC087
ANAC087(315–335)	*At*RST(499–572;R551Q,R560Q)	NB^b^					RST
ANAC087(315–335)	*At*RST(499–572;I563Q)	NB					RST
ANAC087(315–335)	*At*RST(499–572;R560Q)	NB					ANAC087
ANAC087(315–335)	*At*RST(499–572;R551Q)	NB					ANAC087
*Hv*DRF1.1(255–273)	*At*RST(499–572)	450 ± 169	0.95 ± 0.03	−48.7 ± 2.4	12.5	−36.2	RST
*Hv*NAC013(176–346)	*At*RST(499–572)	343 ± 180	1.03 ± 0.04	−4.5 ± 0.3	−32.5	−37.0	RST
*Hv*DRF1.1(255–273)	His_6_-*Hv*RST(467–579)	NB^b^					RST
*At*DREB2A(244–272)	His_6_-*Gm*RST(483–583)	320 ± 72	0.95 ± 0.02	−38.7 ± 1.0	1.6	−37.1	RST
ANAC013(254–274)	His_6_-*Gm*RST(483–583)	526 ± 117	0.94 ± 0.02	−36.7 ± 1.1	0.8	−35.9	RST
*Gm*DREB2A(273–290)	His_6_-*Gm*RST(483–583)	2591 ± 515	0.98 ± 0.04	−23.5 ± 1.1	−8.4	−31.9	RST
*Gm*DREB2A(273–290)	*At*RST(499–572)	397 ± 34	0.85 ± 0.00	−34.9 ± 0.2	−1.6	−36.5	RST

All experiments were performed as described in Methods. Syringe indicates whether RCD1–RST(499–572) or the TF is the titrant (in syringe). The standard errors for Δ*H*, *K*_*d*_ and *N* were obtained from Origin when fitting the data to a ‘one set of sites’ binding model. ^a^Previous data^30^. ^b^No detectable binding (NB).

### Conservation of RCD1-binding in functionally redundant NAC TFs

For the NAC046 TFs, SLiM conservation was less clear than for the other TF groups, with the RBS-V1.{1,2}[^RK][YF].{1,4}[^RK][DE][LI] suggested from the alignment (Fig. 3C; Table 1). Focusing on the NAC046 TFs in *Arabidopsis*, this motif is present in ANAC087 (*At*5g18270) (Fig. 3C), the inparalog most closely related to ANAC046^40^. Both *ANAC046* and *ANAC087* are induced by abiotic stress^40,42^ and these genes redundantly control the onset of cell death execution^47^. ANAC087(315–335) bound *At*RCD1-RST(499–572) with a relatively weak affinity corresponding to *K*_*d*_ 1751 nM (Table 2). This was also the case for ANAC046:RCD1-RST association (*K*_*d*_ 609 nM)^42^ and in both cases entropy significantly contributed to binding. To address if the RST domain uses the same residues for binding of ANAC087 as for *At*DREB2A (Fig. 1D)^25^, binding of ANAC087(315–335) to the double substitution variant *At*RCD1-RST(499–572;R551Q;R560Q) was analyzed. R551 is conserved without having an essential role in the RST-fold, while R560 varies (Fig. 1B,E). No binding was observed for ANAC087 (Table 2) as in the case of *At*DREB2A^25^. The single substitution variants *At*RCD1-RST(499–572;I563Q) and *At*RCD1-RST(499–572;R560Q), with no and compromised *At*DREB2A(255–272) binding, respectively (Fig. 1D)^25^, did not bind ANAC087(315–335). Similar to R551, I563 is chemically conserved despite no structural involvement in the RST fold, thus suggesting that other functional constraints warrant conservation (Fig. 1B,E). In conclusion, ANAC087 binds RCD1 and shares key RCD1-target residues with *At*DREB2A.

### Evolutionary conservation of the RCD1-TF system

**Figure 6:**
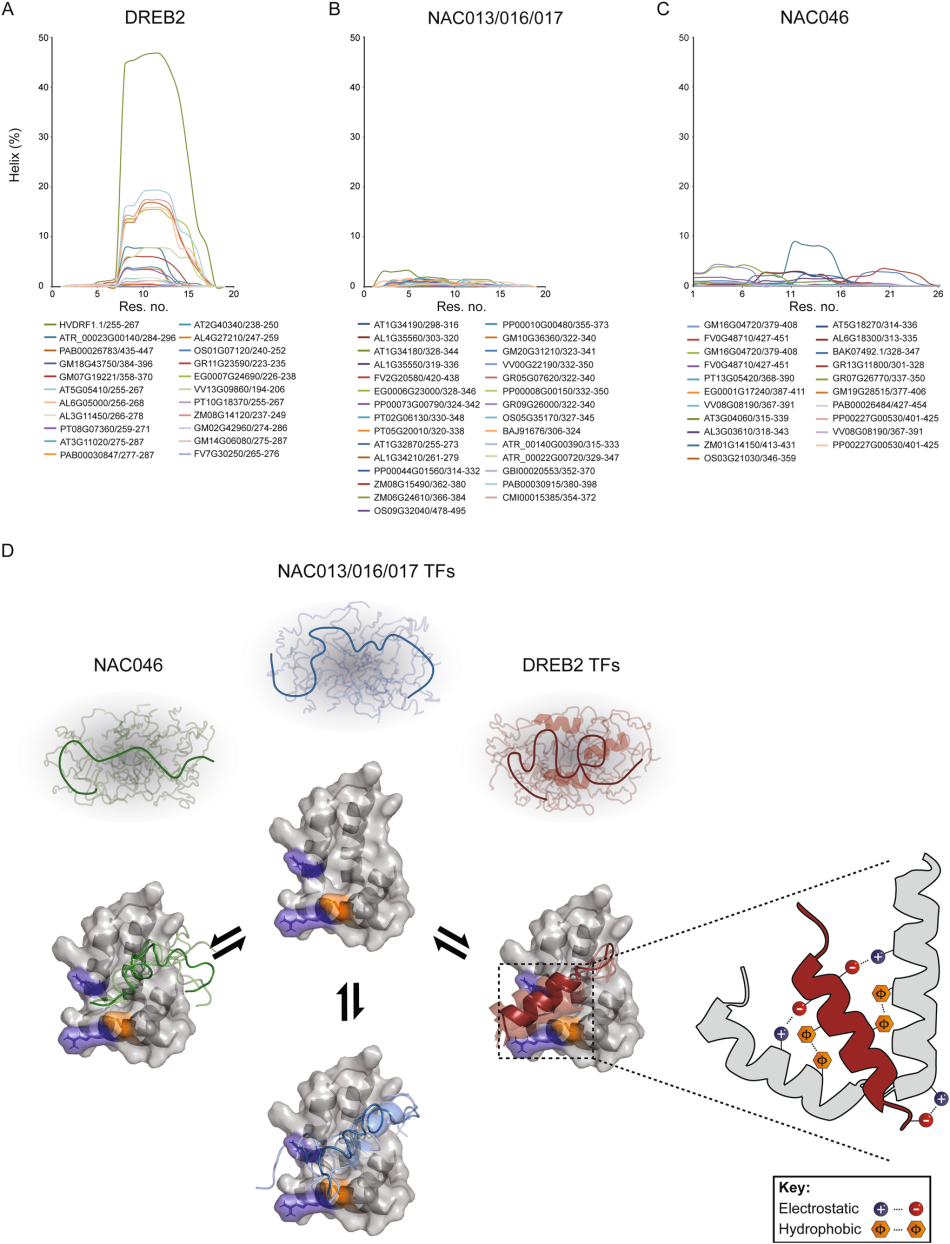
Evolution of the RCD1:TF system. **(A**–**C)** Helicity per residue predicted by Agadir for the DREB2, NAC013/016/017 and NAC046 SLiM regions from different plant species. **(D)** Model of the RCD1:TF interactome based on the structures of the *At*RST domain (PDB 5OAO) and *At*DREB2A(255–272) (PDB 5OAP) from the *At*RCD1-RST:*At*DREB2A complex. The different RCD1 interacting TFs compete for binding to RCD1. The TFs have different helical propensities and may form different structures in their bound states. Complexes with ANAC013 and ANAC046 were modelled using the CABS-dock server. The basic residues R551 and R560 and the hydrophobic residue I563, which are of key importance to TF-binding, are highlighted as blue and orange sticks, respectively. The schematic cartoon displays the typical binding interface between RCD1-RST and the TF activation domains. Activation domains are enriched in acidic and hydrophobic residues that interact with basic and hydrophobic contacts within the RCD1-RST binding pocket. The cartoon is based on the modelled complex between *At*RCD1-RST and *At*DREB2A^25^.

Evolutionary conservation of the RCD1-TF system was also addressed, both by predictions and experimentally. Predictions of the α-helix propensities of the SLiM regions from different species suggested a range of α-helix propensities for the DREB2 TFs (Fig. 6A). Up to 47% α-helix was predicted for the SLiM region in barley *Hv*DRF1.1, and CD experiments suggested a slightly higher intrinsic population of α-helix in *Hv*DRF1.1(255–273) (16%) than in the *At*DREB2A (12%) (Fig. 5B). Several DREB2A SLiM regions have the same level of α-helix propensity as *At*DREB2A (max. 20%), and some SLiM regions such as those from soybean, GM02G42960 and *At*DREB2B and *At*DREB2C, which bound *At*RCD1^30^, have lower α-helix propensities (max 4%). Stress-associated *Hv*DRF1.1^48^ is the only barley DREB2 homolog with a SLiM (Fig. 3B). *Hv*DRF1.1(255–273) bound *At*RCD1-RST(499–572) with *K*_*d*_ 450 nM (Table 2) suggesting that the DREB2A-binding site of a dicot RST domain can accommodate DREB2A from an evolutionary distant monocot. The barley ortholog of ANAC046, *Hv*NAC013(176–346), which in the yeast two-hybrid system bound *Hv*RCD1-RST^49^, also bound *At*RCD1-RST(499–572), further indicating that features of the RCD1-TF interactome are evolutionary constrained. No binding of *Hv*DRF1.1(255–273) to *Hv*His_6_-RCD1-RST(467–579) was detected. This may be explained by the lack of a positively charged residue in the position corresponding to Arg-560 of *At*RCD1 (Fig. 1B,C), which is required for its association with *At*DREB2A^25^. The low α-helix propensities of ANAC013 and ANAC046^30^ are evolutionary conserved among the NAC013/016/017 and NAC046 TFs (Fig. 6B,C).

Conservation of the RCD1:TF system was also analysed by testing for conserved interactions within other species. The RCD1:TF interactome is most conserved in dicots, especially eucalyptus and soybean (*Eucalyptus grandis* and *Glycine max*, respectively) (Figs. 2C, 3), with the SLiM found in all TF groups analysed. The RST domain from the soybean RCD1 ortholog, *Gm*RCD1, in which *At*RCD1 key residues for *At*DREB2A binding are conserved (Fig. 1B), indeed bound both *At*DREB2A(244–272) and ANAC013(254–274) with affinities in the mid-nanomolar range (Table 2). When using one of two soybean DREB2 TFs containing the SLiM (Fig. 3B) in BLAST searches for Arabidopsis proteins, *At*DREB2A appeared as a top hit. The GM02G42960 peptide *Gm*DREB2A(273–290), bound *Gm*RCD1-RST(483–583) with an affinity of *K*_*d*_ approximately 2500 nM and the Arabidopsis RST domain, *At*RCD1-RST(499–572), with an affinity of *K*_*d*_ approximately 400 nM (Table 2).

## Discussion

This study demonstrates that the RBS of the ID-based RCD1:TF interactome arose in the land plant lineage 480 mill. years ago (Fig. 2A) and is evolutionary highly conserved. TF from microorganisms do not contain the SLiM further linking it to interactions with RCD1, also only found in land plants^37,41^. Thus, the RCD1:interactome has evolved to cope with land plant life. Confirming their important functions, 13 of 15 *Arabidopsis thaliana* TFs and their SLiMs are conserved in *Arabidopsis lyrata* (Fig. 3), which cluster with *Arabidopsis thaliana* (Fig. 2C). The SLiM is highly conserved in flowering plants (angiosperms), and for most TF groups the SLiM - especially the expanded [ED].{1,2}[^RK][YF].{1,4}[^RK][DE]([LIVMF]|.L) version - was also traced to the ancient sister seed plant clade, gymnosperms (Fig. 2B). It was also identified in all TF groups, except DREB2 and BBX8, from moss. This likely reflects a different defense system in mosses, although the expression of a couple of AP2/ERF transcription factors is affected by abiotic stress in mosses^50^. Structurally, this could be connected to the charge neutralization of Lys/Arg_537_, Lys/Arg_548_ and Asp_556_ (*At*RCD1 numbering) by substitution with Gln, Gln and Ala, respectively, in the moss RST domain (Fig. 1B). Conservation of the SLiM over a large taxonomic range indicates that it is constrained and therefore functional^51^.

Because of their small size and low sequence complexity, SLiMs easily evolve *de novo*^19^. The RBS was identified in six unrelated TF families^30^ making *de novo* evolution at multiple occasions followed by convergence likely suggestive of common regulatory tasks^52^. Accordingly, the interactions of *At*DREB2A, ANAC013, ANAC016, and ANAC017 with *At*RCD1 result in down-regulation of their target genes^32,33^ pointing to a general regulatory function of the RBS.

The TFs have several conserved sequence regions outside their DBDs (Fig. 2D). Most of these are present in species across different taxa of land plants, but some are specific for angiosperms suggestive of functions related to higher plants. For all TF groups, the SLiM maps to a conserved region and overlaps with an ELM in NAC013/016/017 (A4), bZIP23 (D2) and STO (F3) (Fig. 2D). The degron, DEG_COP1_1, present in the STO TFs, is interesting because it is the site for interactions with the E3 ubiquitin ligase COP1 mediating regulated TF degradation^53^. Overlapping SLiMs reflect the conformational adaptability of IDRs allowing interactions with several distinct binding partners. The ID profiles of NAC013/016/017, DREB2, and NAC046 TFs from different taxa revealed complex patterns but also group-specific characteristic (Fig. 4). That the SLiMs map to dips in the disorder profiles indicate conserved local structure propensities though only a few predicted MoRFs appear to be conserved among the NAC013/016/017, DREB2 and NAC046 TFs (Fig. 4). ID-based dendrograms have been suggested as tools for studies of distant relationships among proteins^43^, and such use is supported by the ID-profiles shown in Fig. 4.

The SLiM is least conserved in the NAC046 TFs. *ANAC087* is expressed by the same abiotic stress factors as *ANAC046*^40^, and the two NAC TFs are functionally redundant^47^. ANAC087(315–335), which contains the SLiM variant RBS-V1 (Table 1; Fig. 3C), was able to associate with *At*RCD1-RST(499–572) (Table 2). Although its RCD1-affinity is relatively low, motif contexts may affect binding affinity significantly^30,42^. Furthermore, the RCD1-interaction partners *AtDREB2A*, *ANAC013*, *ANAC087* and *ANAC046* are induced by various abiotic stress parameters^28,31,40,47^, which will affect *in vivo* binding and competition. Together this suggests that additional NAC046 TFs compete for binding to RCD1.

Based on our results, the SLiM was expanded from [ED].{1,2}[^RK][YF].{1,4}[^RK][DE]L to [ED].{1,2}[^RK][YF].{1,4}[^RK][DE]([LIVMF]|.L). Introducing the Leu264→Ile substitution in *At*DREB2A(244–272), mapping to an internal α-helix position in RCD1-bound DREB2A^25^, did not affect binding affinity significantly, although isoleucine is relatively destabilizing in α-helices. This may be explained by helix capping (Fig. 5A) and thermodynamics (Table 2). Enthalpy contributed less to the affinity of *At*DREB2A(244–272;L264I) than of *At*DREB2A(244–272), suggestive of sub-optimal binding geometry. However, this was compensated by entropy, possible due to less loss in entropy upon binding for the substituted peptide. In contrast, 25-fold decrease in affinity was measured for the corresponding substitution in ANAC013(254–274). The small contribution of entropy to binding of the wildtype peptide was changed into an unfavorable contribution upon substitution, indicating that the conformationally restricted isoleucine may be a disadvantage to a dynamic RCD1:ANAC013 complex^30^. Nonetheless, the SLiM variants are prospectively binding-competent putatively expanding the TF-interactome considerably (Fig. 1C).

Evolutionary conservation of the RCD1:TF interactome was indicated by interspecies cross-binding with Arabidopsis and soybean RCD1-RST and DREB2A being interchangeable interaction partners, and with *Hv*DRF1.1 binding *At*RCD1-RST (Table 2) suggestive of conserved interaction interfaces and properties. However, species-specific co-evolution of the RST:TF interfaces was also apparent from comparison of the *At*DREB2A(244–272) and ANAC013(254–274) association with Arabidopsis versus soybean RCD1-RST. The affinities for the soybean RST domain were lower than for the Arabidopsis domain with 25-fold and 58-fold decreases for *At*DREB2A(244–272) and ANAC013(254–272), respectively. In both cases, this was due to a decrease in binding enthalpy reflecting sub-optimal inter-species interfaces. Such pronounced species-specific co-adaptation was not observed for the *Gm*DREB2A(273–290):*Gm*RCD1-RST(483–583) interaction, which may, however, not represent the best molecular match among their different paralogs. Although our study revealed molecular co-adaptation as exemplified by the high affinity *At*RCD1-RST:*At*DREB2A complex, several parameters, such as SLiM context^30,54^, pH^55^ and partner dynamics^55^ may also influence affinity.

Intriguingly, helix folding-upon-binding was detected for *At*DREB2A, but to a lesser extent for ANAC013 and not at all for ANAC046^30^. Accordingly, the α-helix propensities of the DREB2 SLiM regions were higher than those of the NAC013/NAC016/NAC017 and the NAC046 SLiM regions (Fig. 6A–C), further supportive of convergent evolution. Thus, molecular heterogeneity previously suggested for the RCD1:TF interactions seems to be evolutionary conserved. In contrast, binding to other αα-hub domains, such as the PAH domains of the transcriptional regulator Sin3, fully depend on coupled folding and binding with α-helix formation in the TFs^25,56^. *At*RCD1-RST was able to bind the *Hv*DRF1.1 SLiM, which has a higher intrinsic propensity for α-helix than the other DREB2A SLiM regions (Figs. 5B; 6A). The mechanism of DREB2A association with RCD1, e.g. conformational selection or induced fit, remains unknown. However, the results suggest that pre-formation of α-helix does not hinder complex formation and vice versa may also not be an advantage.

As for TF activation domains binding co-activators, negatively charged and hydrophobic residues are used by TF IDRs for RCD1 binding^30,42,57^. Likewise, the RCD1-RST domain shows characteristics typical of activation domain-interacting domains. Thus, it has a positively charged patch and a hydrophobic binding pocket^58^. The *DREB2A*, *ANAC01*3, and *ANAC046* genes showed similar expression patterns in response to various stress treatments^28^. The question is whether the corresponding proteins share RCD1 binding site despite their different bound conformations or form dynamic complexes (Fig. 6D) as reported for TF interactions with Mediator coactivator subunits^59–61^. RCD1-RST(499–572) mutant analysis (Table 2) indicate that ANAC087 targets the same interface on RCD1 as *At*DREB2A^25^ despite its low α-helix propensity (Fig. 6C) and may explain why Arg551 and Ile563 are conserved without contributing to the RST core structure (Fig. 1B,E). Interestingly, *Hv*RCD1 has a valine in the position corresponding to Arg560 of *At*RCD1 (Fig. 1B), which is important for TF-binding, and *Hv*RCD1 did not bind *Hv*DRF1.1 (Table 2). Furthermore, both electrostatic and hydrophobic interactions are involved in RCD1 interactions with the DREB2A and NAC TFs^25,30,46^ (Table 2). Together these data lead to the current RCD1:TF binding model (Fig. 6D) which points out the structural plasticity of the RCD1:TF interactome. Additionally, this whole-interactome study opens new scientific avenues addressing co-evolution of structure and dynamics in interactomes, evolution-based interactome expansion, ID:hub interaction mechanisms based on whole-interactome strategies, and *in vivo* competition for TF binding to RCD1.

## Methods

### Bioinformatics

Inparalogs and orthologs were identified using Integrative Orthology Viewer at PLAZA, which integrates four complementary methods^34,35^. Dicots PLAZA 4.0, Monocots PLAZA 4.0, Gymno PLAZA 1.0, and Pico-PLAZA 2.0 were used to obtain data from a wide range of plant species. The protein sequences are named by the PLAZA identifier codes which are specific for the Dicot, Monocot and Gymno PLAZA platforms. In addition, BLAST homology searches^62^ were performed to obtain full-length proteins from *Hordeum vulgare* (barley). The WWE (PS50918) and PARP (PS51059) domains were identified by ScanProsite^63^ which scans the submitted sequences against the signatures from the Prosite database. The RST domain (PF12174) was identified by SMART^64^ in the ‘normal’ mode which allows SMART to search for domains by scanning the homologues sequences against the whole SMART database. In addition to the protein domains annotated in SMART, the search for Pfam domains was included. Multiple protein sequence alignments were created using Clustal Omega^65^ and evaluated using Transitive Consistency Score. For the multiple sequence alignments, minor manual adjustments were made in Jalview^45^. The TFs were analyzed for ID-conservation using IUPRED^66^, which allows disorder prediction for multiple sequences imported from Jalview. The median as well as the first and third quartiles were calculated for the IUPRED scores at each alignment position in order to show the interquartile range together with minimum and maximum values. Eukaryotic Linear Motif (ELM)^67^ was used to search for documented eukaryotic SLiMs, and α-helix propensity was analyzed using Agadir^68^. MoRFpred^69^ was used for prediction of MoRFs counting four or more residues. The non-redundant protein database was searched for additional transcription factors putatively binding RCD1 using two different BLAST programs, PHI-BLAST and PSI-BLAST^62^.

### Modelling

The structures of *Hv*RST, *Gm*RST and *Pp*RST were modelled using the I-TASSER^70^ webserver (https://zhanglab.ccmb.med.umich.edu/I-TASSER/) with standard settings and no constraints. Complexes between *At*RST and either ANAC013 or ANAC046 were modelled using the CABS-dock server^71^ (http://biocomp.chem.uw.edu.pl/CABSdock/), with the structure of complexed *At*RST (PDB 5OAO) and the peptide sequences of ANAC013(254–274) or ANAC046(319–338) as input.

### Cloning, heterologous expression and protein purification

The cDNA encoding *Glycine max* (soybean) RCD1 (ID XP_003516978) residues 483–583 was obtained from ATUM and cloned into pET-15b. The RST domain was purified as previously described for RST domains^42,49^. ANAC087 (ID AT5G18270) and *At*DREB2A (ID AT5G05410) cDNAs were obtained from the REGINA collection (Paz-Ares and the REGIA Consortium 2002). Gene-specific primers, encoding a tobacco etch virus cleavage site, were used for PCR-based amplification and the fragments cloned into pGEX-4T-1 (GE Healthcare) to produce GST-tagged proteins. The constructs were verified by sequencing, and site-specific mutations were introduced using the QuikChange mutagenesis kit (Stratagene). The GST-tagged fusion proteins were expressed in *Escherichia coli* strain BL21-(DE3) at 37 °C, induced by 0.5 mM isopropyl-D-thiogalactopyranoside, harvested after 3 h, resuspended in 20 mM Tris-HCl, pH 8.0, 100 mM NaCl, and sonicated. After centrifugation for 15 min at 36.000 × *g*, the supernatant was incubated at 4 °C for one h with glutathione-Sepharose 4B (Sigma) resin. Bound GST-tagged recombinant protein was cleaved from the GST tag using tobacco etch virus protease by incubation overnight in resuspension buffer containing 1 mM EDTA, 5 mM DTT and 1.0 µg of tobacco etch virus/0.1 mg protein. To remove the protease after cleavage, the disordered fragments were heated at 72 °C for 10 min and centrifuged at 20,000 × *g* for 10 min. Salt was removed from the peptides by freeze-drying before resuspension in 0.1% trifluoroacetic acid and purification on a Vydac C18 column (Grace) equilibrated in 20% ethanol, 0.1% trifluoroacetic acid and eluted in a linear gradient from 20 to 100% ethanol. Purified peptides were analyzed by MALDI-TOF (Autoflex Bruker) mass spectrometry and SDS-PAGE. N-terminally acetylated and C-terminally amidated peptides of *Hv*DRF1.1(255–273) (ID AAO27885.1) and *Gm*DREB2A(273–290) (ID XP_028217593.1/PLAZA ID GM02G42960) were obtained from TAG Copenhagen A/S. Plasmids encoding ANAC013 (ID AT1G32870) residues 254–274, *Hv*NAC013 (ID AK376297.1) residues 176–346, and *Hv*RCD1 (ID FR846236) residues 467–579 were from previous work, and the peptides and protein were expressed and purified as described^30,49^.

### CD spectroscopy

Far-UV CD spectra of peptides were recorded in 10 mM Na_2_HPO_4_/Na_2_PO_4_, pH 7.0, at 15–20 µM and increasing amounts of TFE (0–40%; v/v) as indicated in the figure legends. Details of the recordings were as in^42^.

### Isothermal titration calorimetry (ITC)

ITC was used to determine the thermodynamic parameters, the dissociation constant (*Kd*), the stoichiometry (*N*), and the binding enthalpy change (Δ*H*) from which the binding Gibbs free energy change (Δ*G*) and the binding entropy change (Δ*S*) were calculated. The experiments were performed in a MicroCal ITC200 microcalorimeter (GE Healthcare). Protein samples were dialyzed against 50 mM Hepes, pH 7.4, 100 mM NaCl, centrifuged at 20,000 × *g* for 10 min, and degassed for 10 min by stirring under vacuum. The protein concentration in the sample cell was 10–30 µM and the titrant concentrations in the syringe were 100–300 µM. A total of 18 injections separated by 180 s and with a duration of 4 s each of 2 µl titrant was injected into the sample at 25 °C. The Origin 7 software package (MicroCalTM) was used for fitting the data to a “one set of sites” binding model. Standard errors for Δ*H, Kd*, and *N* were obtained from Origin when fitting the data. All experiments were repeated at least three times. The heat of dilution was subtracted from the raw data by performing a titration of titrant against buffer or by subtracting the dilution enthalpy obtained when the enthalpy change had reached a constant level.

## Supplementary information

Supplementary information 
