# Supplementary material for: Evolutionary conservation of the intrinsic disorder-based Radical-Induced Cell Death1 hub interactome
Source: Sci Rep. 2019 Dec 12;9:18927. doi: 10.1038/s41598-019-55385-3 (PMC6908617; doi:10.1038/s41598-019-55385-3)
Supplement: Supplementary file 1 — Supplementary information [file 41598_2019_55385_MOESM1_ESM.pdf]

Supplementary Information

Evolutionary conservation of the intrinsic disorder-based Radical-Induced Cell Death1 hub interactome

Lise Friis Christensen[1], Lasse Staby[1], Katrine Bugge[1], Charlotte O'Shea[1], Birthe B. Kragelund[1] and Karen Skriver[1]

[1]REPIN and the Linderstrøm-Lang Centre for Protein Science, Department of Biology, University of Copenhagen, Copenhagen DK-2200, Denmark
Correspondence should be addressed to K.S. (email: kskriver@bio.ku.dk)

Supplementary Table S1. Classification of SRO-protein containing plant species examined in this study.

Supplementary Table S2. SRO-proteins in the plant species examined in this study.

Supplementary Table S3. PHI-BLAST searches for putative RCD1-interacting TFs.

Supplementary Figure S1. Conservation of the RBS.

**Classification**			**Family**	**Species**	**ID**	**Common name**
Bryophytes (non-vascular)			Funariaceae	*Physcomitrella patens*	pp	Moss
Tracheophytes (vascular)	Lycophytes		Selaginellacae	*Selaginella moellendorffii*	sm	Spikemoss
	Angiospermae		Amborellaceae	*Amborella trichopoda*	atr	-
		Monocot	Poaceae	*Oryza sativa ssp. japonica*	os	Rice
				Zea mays	zm	Maize
				Hordeum vulgare	hv	Barley
		Dicot	Brassicaceae	*Arabidopsis lyrata*	al	-
				Arabidopsis thaliana	at	Thale cress
			Myrtaceae	*Eucalyptus grandis*	eg	Eucalyptus
			Rosaceae	*Fragaria vesca*	fv	Wild strawberry
			Fabaceae	*Glycine max*	gm	Soybean
			Malvaceae	*Gossypium raimondii*	gr	Cotton
			Salicaceae	*Populus trichocarpa*	pt	Western balsam poplar
			Vitaceae	*Vitis vinifera*	vv	Grapevine
	Gymnospermae		Cycadaceae	*Cycas micholitzii*	cmi	-
			Ginkgoaceae	*Ginkgo biloba*	gbi	Maiden hair
			Gnetaceae	*Gnetum montanum*	gmo	-
			Pinaceae	*Picea abies*	pab	Norway spruce

Supplementary Table S1. Classification of SRO-protein containing plant species examined in this study.

Classification			Family	Species	Common name/Identifier (ID)
Bryophytes (non-vascular)			Funariaceae	*Physcomitrella patens*	PpSRO1a/Pp1s341_28V6.1<br>PpSRO1b/Pp1s215_49V6.1<br>PpSRO1c/Pp1s30_104V6.2
Tracheophytes (vascular)	Lycophytes		Selaginellacea	*Selaginella moellendorffii*	SmSRO1a/C_860065
	Angiospermae		Amborellacea	*Amborella trichopoda*	ATR_00024G00230[a]<br>ATR_00048G00660[a]<br>ATR_00056G01400[a]<br>ATR_00058G01980[a]
		Monocot	Poaceae	*Oryza sativa ssp. japonica*	OsSRO1a/Os10g42710[a]<br>OsSRO1b/Os03g63770[a]<br>OsSRO1c/Os03g12820[a]<br>OsSRO1d/Os06g13860[a]<br>OsSRO1e/Os04g57640[a]
				Zea mays	ZM01G08710[a]<br>ZM01G22650[a]<br>ZM05G00270[a]<br>ZM05G12830[a]
			Poaceae	*Hordeum vulgare*	HVU0040G0972[a]<br>RCD1/HVU0041G0124<br>HVU0045G2681[a]
		Dicot	Brassicaceae	*Arabidopsis lyrata*	AL1G37310<br>AL1G46370<br>AL2G29810<br>AL4G31560<br>AL5G26990<br>AL8G39410
				Arabidopsis thaliana	AtRCD1/At1g32230<br>AtSRO1/At2g35510<br>AtSRO2/At1g23550<br>AtSRO3/At1g70440<br>AtSRO4/At3g47720<br>AtSRO5/At5g62520
			Myrtaceae	*Eucalyptus grandis*	EgSRO1a/ Eucgr.E00230<br>EgSRO1b/ Eucgr.F00472<br>EgSRO2a/ Eucgr.B00305<br>EgSRO2b/ Eucgr.B00313<br>EgSRO2c/ Eucgr.B00314
			Rosaceae	*Fragaria vesca*	FvSRO1a/ gene13738-v1.0-hybrid<br>FvSRO1b/ gene13736-v1.0-hybrid<br>FvSRO2a/ gene16492-v1.0-hybrid<br>FvSRO2b/ gene24121-v1.0-hybrid<br>FvSRO2c/ gene27267-v1.0-hybrid
			Fabaceae	*Glycine max*	GmSRO1a/ Glyma09g34000<br>GmSRO1b/ Glyma01g01900<br>GmSRO2a/ Glyma08g12963<br>GmSRO2b/ Glyma05g02210<br>GmSRO2d/ Glyma04g35560
			Malvaceae	*Gossypsium raimondii*	GrSRO1a/ Gorai.004G182400<br>GrSRO1b/ Gorai.008G224200<br>GrSRO1c/ Gorai.009G278000<br>GrSRO1d/Gorai.009G224400<br>GrSRO2a/Gorai.008G295300<br>GrSRO2b/Gorai.008G158900
			Salicaceae	*Populus trichocarpa*	PtSRO1a/Potri.003G096700<br>PtSRO1b/Potri.001G137200<br>PtSRO1c/Potri.002G112300<br>PtSRO2b/Potri.006G231600<br>PtSRO2c/Potri.006G231100<br>PtSRO2d/Potri.006G231500<br>PtSRO2e/Potri.015G076500<br>PtSRO2f/Potri.012G081100
			Vitaceae	*Vitia vinifera*	VvSRO1a/ GSVIVG01013086001<br>VvSRO1b/ GSVIVG01013090001<br>VvSRO2a/ GSVIVG01013740001<br>VvSRO2b/ GSVIVG01007754001
	Gymnospermea		Cycadaceae	*Cycas micholitzii*	CMI00005144<br>CMI00008850
			Ginkgoacea	*Ginko biloba*	GBI00022997
			Gnetacea	*Gnetum montanum*	[b]
			Pinaceae	*Picea albies*	PAB00006078<br>PAB00047409

Supplementary Table S2. SRO-proteins in the plant species examined in this study.

[a]All SRO proteins were identified previously except those marked with a which were identified from PLAZA searches in this study.

[b]None identified.

**ERF/AP**	**WRKY**	**MADS**	**ZF**	**NAC**	**bHLH**	**HD**	**bZIP**	**B3**	**Other**
AT1G01250<br>ERF023	AT1G29280<br>WRKY65	AT3G18650<br>AGL103	**AT1G06040<br>STO**	AT1G01010<br>NTL10	AT1G09530<br>PIF3	AT1G20280	AT1G06070	AT1G19220<br>ARF19	AT1G55650<br>HMGB11
AT1G01840<br>SNZ	AT1G5022<br>WRKY10	AT3G54340<br>AP3	AT1G25440<br>BBX15	AT1G02210<br>NAC	AT2G43010	AT1G23380<br>KNAT6	AT1G39050<br>bZIP60	AT1G28300<br>LEC2	AT1G56170<br>NF-YC2
AT1G16060<br>ARIA	AT1G55600<br>WRKY10	AT5G15800<br>AGL2	AT1G43280<br>BBX17	AT1G32510<br>ANAC011	AT3G19860<br>bHLH121	AT1g62360<br>BUM1	AT1G42990<br>BZIP60	AT1G35520<br>ARF15	AT3G20770<br>EIN3
AT1G22190<br>Rap2.4	AT2G34830<br>WRKY35	AT5G51870<br>AGL71	AT1G43860	AT1G32770<br>SND1	AT3G47640<br>PYE	AT1G64570<br>DUO3	AT1G58110	AT1G50220	AT5G05330
AT1G25470<br>CRF12	AT3G58710<br>WRKY69	AT5G27130<br>AGL39	AT1G49130<br>BBX17	**AT1G32870<br>ANAC013**	AT3G59060<br>PIF5	AT1G66230<br>MYB20	AT2G17770<br>bZIP27	AT2G18810	AT5G35680
**AT1G36060<br>RAP2.4A**	**AT4G01720<br>WRKY47**		AT1G49900	**AT1G34180<br>ANAC016**	**AT4G00050<br>UNE10**	AT1G70510<br>KNAT2	AT2G40620<br>bZIP18	AT2G24700	
AT1G49120<br>CRF9	AT5G45260<br>ATWRKY52		AT1G60250<br>BBX26	**AT1G34190<br>ANAC017**	AT4G14410<br>BHLH104	AT1G79180<br>MYB63	AT2G40950<br>BZIP17	AT3G24650<br>ABI3	
AT1G63030<br>DREB1A	AT5G46310		AT1G62220<br>BBX27	AT1G69490<br>NAP	AT4G28815<br>BHLH127	AT2G30380	AT3G10800<br>bZIP28		
AT1G68550<br>CRF10			AT1G62310<br>jmjC	AT2G27300<br>NTL8	**AT4G36060<br>BHLH11**	AT2G31180<br>MYB14	AT3G49760<br>bZIP5		
AT1G71450<br>FUF1			AT1G68190<br>BBX27	AT3G01600<br>ANAC082		AT2G32370<br>HDG3	AT3G56660<br>BZIP49		
AT1G77200			AT1G68520<br>BBX14	**AT3G04060<br>ANAC046**		AT3G16350<br>HD	AT4G35040<br>bZIP19		
AT2G22200			AT1G78600<br>LZF1	AT3G10500<br>NTL4		AT3G60460<br>DUO1	AT5G06839<br>BZIP65		
AT2G24681			AT2G20180<br>TZF4	AT3G43660<br>NTL6		AT4G08150<br>KNAT1			
AT2G33710			AT2G21830	AT3G44290<br>NTL5		AT4G17060<br>FIP1			
AT2G35700<br>ERF38			AT2G24790<br>COL3	AT3G49530<br>ANAC062		AT4G18770<br>MYB98			
**AT2G40340<br>DREB2C**			AT2G31380<br>BBX25	AT4G01550<br>ANAC069		AT5G03680<br>PTL			
AT2G43920<br>RAP2.2.			AT2G33835<br>FES1	AT4G01970<br>NTM2		AT5G11050<br>MYB64			
AT2G44940			AT2G45050<br>GATA2	AT4G10230		**AT5G11270<br>OCP3**			
AT2G46310<br>CRF5			AT3G07650<br>COL9	AT4G35580<br>CBNAC		AT5G38160<br>MYB22			
AT3G16280			AT3G24050<br>GATA1	AT5G03830<br>ANAC077		AT5G40430<br>MYB22			
AT3G16770<br>RAP2.3			AT3G29765<br>GTF2	**AT5G09330<br>VNI1**		AT5G44180<br>RTL2			
AT3G20310<br>ARF7			AT3G60530<br>GATA4	AT5G22290<br>ANAC089		AT5G44190<br>GLK2			
AT3G23240<br>ERF1			AT3G63430<br>TRM5	AT5G24590<br>TIP		AT5G46880<br>HDG5			
AT3G25890<br>CRF11			AT4G15080	AT5G39690<br>ANAC093		AT5G49330<br>MYB111			
AT3G53310			AT4G16845<br>VRN2	AT5G64060<br>ANAC103					
AT3G54320<br>WRI1			AT4G36240<br>GATA7						
AT3G57600			AT5G24930<br>COL4						
AT3G60490			AT5G25830<br>GATA12						
AT3G61630<br>CRF6			AT5G43630<br>TZP						
AT4G11140<br>CRF1			**AT5G48250<br>BBX8**						
AT4G13040			AT5G57660<br>COL5						
AT4G16750<br>ERF039			AT5G66320<br>GATA5						
AT4G23750<br>CRF2									
AT4G27950<br>CRF4									
AT4G31060<br>RAP2.1									
**AT5G05410<br>DREB2A**									
AT5G17430<br>Baby Boom									
AT5G25810									
AT5G65130<br>WIND4									
AT5G67000									

Supplementary Table S3. PHI-BLAST searches for putative RCD1-interacting TFs. [ED].{1,2}[^RK][YF].{1,4}[^RK][DE]([LIVMF]|.L) was used as regular expression for the searches. The putative binders are listed according to TF families. TFs which contain a SLiM and were previously shown to bind RCD1 are shown in bold.

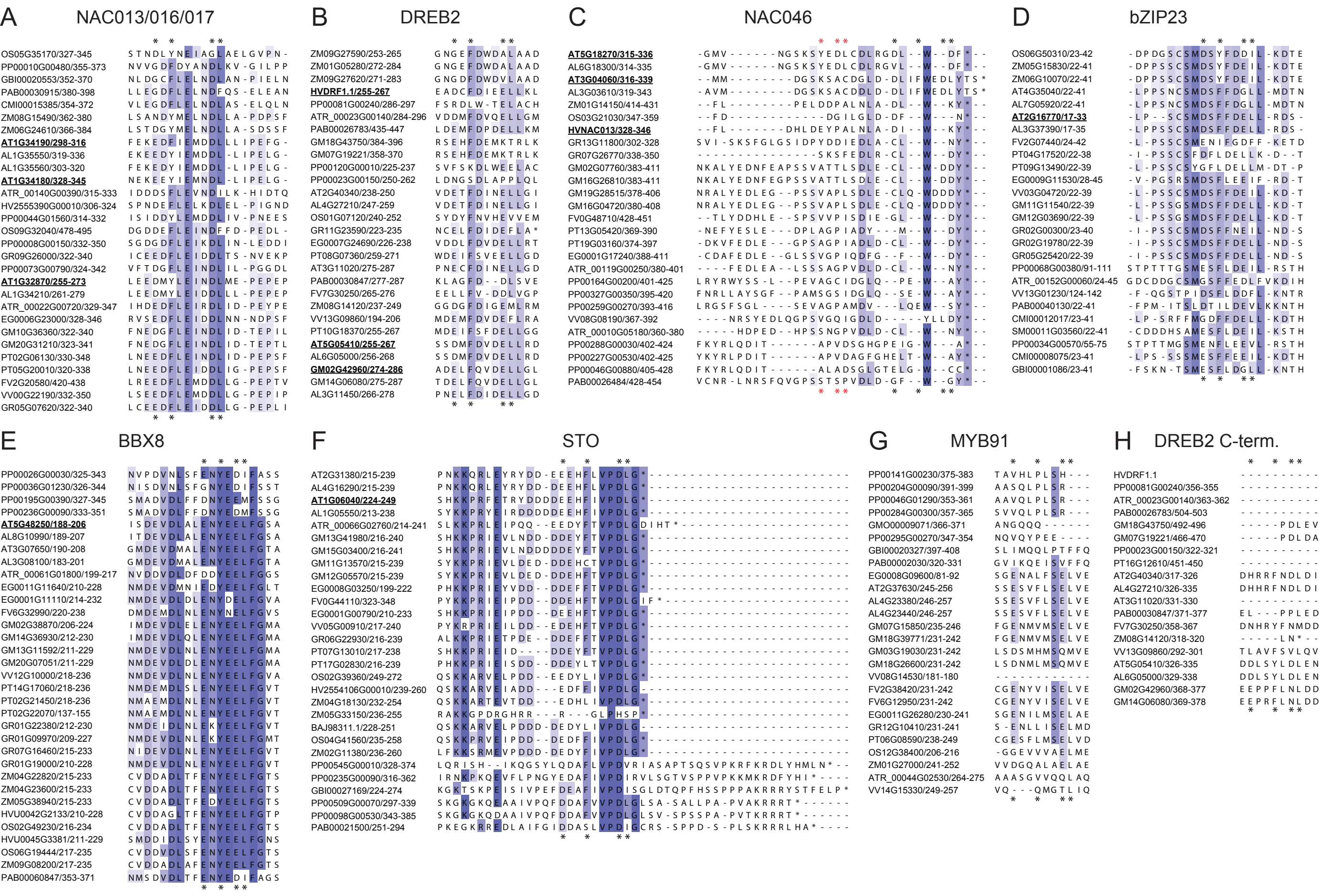
A NAC013/016/017
B DREB2
C NAC046
D bZIP23
E BBX8
F STO
G MYB91
H DREB2 C-term.

Supplementary Figure S1. Conservation of the RBS. **(A-F)** Sequence alignments based on the experimentally verified RBS from the Arabidopsis TFs ANAC013 (AT1G32870), ANAC016 (AT1G34180), ANAC017 (AT1G34190), *At*DREB2A (AT5G05410), *At*DREB2B (AT3G11020), *At*DREB2C (AT2G40340), ANAC046 (AT3G04060), *At*bZIP23 (AT2G16770), *At*BBX8 (AT5G48250), and *At*STO (AT1G06040) with homologs from different species. The gene names of experimentally verified binders of *At*RCD-RST are underlined. The aligned regions do not represent the exact constructs used in the experimental studies. The figure shows the names without the prefix A or *At*, specifically referring to NAC TFs of *Arabidopsis* origin, since sequences from many different plant species are shown. The amino acid residues are coloured according to the degree of conservation (Jalview; darker blue is more conserved). For all alignments, SLiM residues are marked with black asterisks in the top and bottom of the alignment. For ANAC087 (AT5g18270), the alternative putative SLiM variant is marked with red asterisks. **(G-H)** Sequence alignment of regions corresponding to non-binding SLiMs from *At*MYB91 (AT2G37630) and *At*DREB2A (C-terminal SLiM). The sequences were obtained from the PLAZA platform for the following species: *Physcomitrella patens* (PP), *Selaginella moellendorffii* (SM), *Amborella trichopoda* (ATR), *Oryza sativa ssp. Japonica* (OS), *Zea mays* (ZM), *Arabidopsis lyrata* (AL), *Arabidopsis thaliana* (AT), *Eucalyptus grandis* (EG), *Fragaria vesca* (FV), *Glycine max* (GM), *Gossysium raimondii* (GR), *Populus trichocarpa* (PT), *Vitis vinifera* (VV), *Ginkgo biloba* (GBI), *Cycas micholitzii* (CMI), *Gnetum montanum* (GMO) and *Picea abies* (PAB). The sequences of the plant species *Hordeum vugare* (HV, BAJ or AAO) were obtained by BLAST homology searches.
